# Root System Architecture and Abiotic Stress Tolerance: Current Knowledge in Root and Tuber Crops

**DOI:** 10.3389/fpls.2016.01584

**Published:** 2016-11-01

**Authors:** M. A. Khan, Dorcus C. Gemenet, Arthur Villordon

**Affiliations:** ^1^International Potato CenterLima, Peru; ^2^Louisiana State University Agricultural Center, ChaseLA, USA

**Keywords:** root system architecture (RSA), abiotic stress tolerance, root and tuber crops, drought tolerance, sweetpotato, potato, yam, cassava

## Abstract

The challenge to produce more food for a rising global population on diminishing agricultural land is complicated by the effects of climate change on agricultural productivity. Although great progress has been made in crop improvement, so far most efforts have targeted above-ground traits. Roots are essential for plant adaptation and productivity, but are less studied due to the difficulty of observing them during the plant life cycle. Root system architecture (RSA), made up of structural features like root length, spread, number, and length of lateral roots, among others, exhibits great plasticity in response to environmental changes, and could be critical to developing crops with more efficient roots. Much of the research on root traits has thus far focused on the most common cereal crops and model plants. As cereal yields have reached their yield potential in some regions, understanding their root system may help overcome these plateaus. However, root and tuber crops (RTCs) such as potato, sweetpotato, cassava, and yam may hold more potential for providing food security in the future, and knowledge of their root system additionally focuses directly on the edible portion. Root-trait modeling for multiple stress scenarios, together with high-throughput phenotyping and genotyping techniques, robust databases, and data analytical pipelines, may provide a valuable base for a truly inclusive ‘green revolution.’ In the current review, we discuss RSA with special reference to RTCs, and how knowledge on genetics of RSA can be manipulated to improve their tolerance to abiotic stresses.

## Introduction to Roots and Root System Architecture

Roots are essential for plant productivity and serve a variety of functions, such as water and nutrient uptake, forming symbioses with other microorganisms in the rhizosphere, anchoring the plant to the soil, and acting as storage organs. The different interactions of a root with its environment depend on its organization and structure, from the cellular to whole-plant level. The root contains a stele, comprised of the xylem, the phloem, and the pericycle (Smith and De Smet, 2012). The stele is encircled by concentric layers of epidermal, cortical, and endodermal tissues. The root apical meristem forms the basic stem cell pool from which other cell types develop. This root apical meristem also holds the quiescent center (QC), with rarely dividing cells that signals the surrounding cells to organize and maintain the initial stem cells (Dolan et al., 1993). There are generally two types of roots: (i) those that are formed in the embryo, such as the primary and seminal roots in maize (Hochholdinger, 2009), tap or primary root in common bean (Lynch and Brown, 2012); (ii) those formed post-embryonically from consecutive nodes on shoots, normally referred to as adventitious roots (ARs). These include basal roots in beans, nodal roots in maize, ARs of sweetpotato, potato, cassava, as well as yam, and lateral roots (LRs; Lynch and Brown, 2012). LRs are formed post-embryonically from the pericycle of all root classes through auxin-dependent cell cycle activation. This cell cycle forms the LR founder cells that undergo several rounds of cell division to initiate LRs (Overvoorde et al., 2010). The elongation, growth angles from the main axis, lateral branching and longevity of all root classes forms the root system which is determined by genetic, physiological, and environmental factors (Lynch and Brown, 2012).

Root system architecture (RSA) therefore refers to the spatial configuration of the root system or the explicit deployment of root axes (Lynch, 1995). Under poorly understood genetic control, RSA exhibits plasticity and responds to external environmental conditions such as soil moisture, nutrients, temperature, pH, and microbial communities (Bao et al., 2014). The study of RSA is important for agricultural productivity because most soils have uneven distribution of resources and/or localized depletions that make spatial distribution of the root system an important determinant of a plant’s ability to exploit these resources (Lynch, 1995). Progress in the study of RSA in agricultural crops has consequently been realized, especially for cereals, and evidence for the genetic control of RSA and its relationship to increased productivity under stress is currently well-documented. Despite these achievements, information on RSA in root and tuber crops (RTCs), which form the second largest group of crops for global food security after cereals, is still lacking. A recent review by Villordon et al. (2014b) on root architecture and RTC productivity clearly indicates this gap. In the current review, we discuss RSA with special reference to RTCs, the genetic control of RSA, the relationship between RSA and abiotic stresses, and how RSA can be manipulated to confer tolerance to abiotic stresses. We then draw conclusions on the way forward for RSA studies in RTCs.

## Root System Architecture in Root and Tuber Crops

**FIGURE 1:**
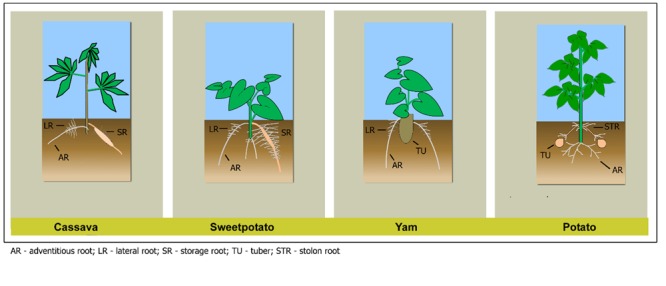
**Root system architecture of cassava, sweetpotato, yam, and potato showing different root types (potato and sweetpotato figures adapted from Villordon et al., 2014b)**.

Understanding RSA and the mechanisms of its development will allow manipulation and exploitation of different root traits to improve plants’ adaptation to changing climates and increase yields for the growing global human population (Smith and De Smet, 2012). Vegetatively propagated RTCs such as potato (*Solanum tuberosum*), sweetpotato (*Ipomoea batatas*), cassava (*Manihot esculenta*), and yam (*Dioscorea* spp.) provide food security for vast populations, especially in sub-Saharan Africa where many resource-poor small holder farmers provide the majority of food. Of the four major RTCs, cassava and sweetpotato are storage roots, while potato and yam are tubers. Little literature is specifically targeted to root growth and development in RTCs compared to cereals. The little literature available also mainly focuses on the growth and development of the storage root or tuber, primarily at harvest and post-harvest evaluations, rather than the entire root system. In this section we describe the root systems in the four major RTCs, based on available literature (**Figure 1**).

Cassava, potato, sweetpotato, and yam have ARs originating from the shoot or subterranean stem, in contrast with the primary root in seed-propagated crops which originates from the embryo. In sweetpotato and cassava, RSA is composed of ARs, LRs and storage roots (SRs), whereas in potato, the ARs can be divided into basal (ARs in **Figure 1**) and stolon roots (STR). In yam, the ARs root system is the most pronounced. The simple recognition of the main AR axis and its spatial and temporal relationship to LRs and their initiation in RTCs would enable systematic investigations to further understand the mechanisms that trigger LR emergence and morphogenesis.

### Root Architecture in Root and Tuber Crops: The Current State of Knowledge

A comparative survey of reports published in the last 25 years on the subject of root architecture among cassava, potato, sweetpotato, and yam is shown in **Table 1**. In general, current knowledge is at the level of classical morphology, with relatively little on the genetic, hormonal, and molecular control of root architecture development among RTCs. The first available documented attempt to specifically describe RSA development of several vegetable species, including sweetpotato, across different developmental stages was by Weaver and Bruner (1927). In sweetpotato, the pericyclic development of LRs and its connection to protoxylem poles, where the number of protoxylem poles correspond to the number of LRs on enlarged storage roots, was made in the early 1900s (Hayward, 1938). Yasui (1944) later reported that the protoxylem in ARs of sweetpotato was generally either pentarch or hexarch, and that adventitious buds arose from five or six longitudinal rows of LR “scars.” Relatively recent work documenting the pericyclic origin of cassava LRs noted that xylem poles ranged from four to eight and LRs developed from the pericycle opposite the xylem pole (Medina et al., 2007; Bonfim et al., 2011). Chaweewan and Taylor (2015) found that the roots developing from stem cut end of cassava (basal ARs) did not develop into storage roots. Such roots were also initiated from the cambium. However, roots developing from buried nodes (nodal ARs) at the boundary between the xylem and the stele had the capacity to develop into storage roots. Only one reference was found for yams, the second most important root crop in Sub-Saharan Africa^1^, which described two distinct well-organized root systems: the seminal root system and the adventitious, more definitive root system (Charles-Dominique et al., 2009). Other studies in yam only focused on root morphology at crop harvest stage in response to fertilization (Melteras et al., 2008; O’Sullivan, 2008; Hgaza et al., 2012). Iwama et al. (1977) specifically analyzed root systems and the relationship between root systems and tuber yield in potato (Iwama et al., 1981). The effect of environment on RSA has been examined from different angles, for example Asfary et al. (1983) measured average root length under different nitrogen (N) fertilization, Vos and Groenwold (1986) studied root growth of potato on a marine clay soil, while Parker et al. (1989) studied the properties of subsoil loosening and irrigation on soil physical characteristics, root distribution, and water uptake in potato. More recently, Iwama (2008) studied the physiology and morphology of potato roots, specifically root length distribution, and examined their relationship with tuber growth.

**Table 1:** Summary of articles published within the last 25 years that address root architecture development in cassava, sweetpotato, potato, and yams.

Subject	Crop species	Reference
Morphological description	Potato	Wishart et al., 2013
	Cassava	El-Sharkawy, 2004
	Sweetpotato	None found
	Yam	Charles-Dominique et al., 2009
Functional anatomy	Potato	None found
	Cassava	Bonfim et al., 2011
	Sweetpotato	None found
	Yam	None found
Genetic and hormonal control	Potato	Xie et al., 2011; Roumeliotis et al., 2012
	Cassava	None found
	Sweetpotato	Ku et al., 2008
	Yam	None found
Environmental signals	Potato	Dechassa et al., 2003; Busse and Palta, 2006; Palta, 2010
	Cassava	Pardales and Esquibel, 1996; Pardales and Yamauchi, 2003; Subere et al., 2009
	Sweetpotato	Pardales and Yamauchi, 2003; Villordon et al., 2012, 2013
	Yam	None found
Breeding	Potato	Iwama, 2008; Wishart et al., 2013
	Cassava	Pardales and Yamauchi, 2003
	Sweetpotato	Pardales and Yamauchi, 2003
	Yam	None found

### The Link between Root Architecture and Yield in Root and Tuber Crops

In sweetpotato, storage root formation is marked by the formation of cambia around the protoxylem and secondary xylem elements, but lignification in the stele region reduces storage root formation (Togari, 1950; Wilson and Lowe, 1973). The first evidence of a link between sweetpotato LR development and storage root yield suggested that LRs may be essential in supplying “internal growth elements” for storage root formation (Koshimizu and Nishida, 1949). Recent work demonstrated the link between LR development and lignification. In ARs with a prevalence of arrested or non-emerged LR primordia, the adjacent stelar tissue becomes lignified thus rendering it incapable of undergoing swelling due to the absence of vascular and anomalous cambia development (Villordon et al., 2012). The precise relationship between stele lignification and LR development is still not clear in sweetpotato. However, proteomics work with maize *lrt1* (lateral rootless1) mutants showed the detection of proteins associated with lignin metabolism in the primary root, providing evidence that LRs influenced the proteome of the primary root (Hochholdinger et al., 2004). These findings suggest that intrinsic and external stimuli which promote LR development preclude stele lignification, rendering the juvenile AR competent for storage root formation. In cassava, LRs are responsible for root system plasticity during the critical storage root formation stage (Pardales and Yamauchi, 2003). There is currently a lack of evidence to suggest a relationship between LR development and the capacity of an AR to become a storage root. Early work describing anatomical changes associated with storage root formation in cassava did not mention LRs (Indira and Kurian, 1977). Related work examined the branching pattern of LRs and reported that LRs increased root surface area and compensated for the decrease in the main root length (Izumi et al., 1999). It was concluded that roots with a well-developed branching pattern likely absorbed water and essential nutrients for storage root growth better. In potato, root mass is positively correlated with shoot mass and tuber bulking, but negatively correlated with early tuber bulking. Final tuber yield is related to RSA component traits such as specific root length of basal roots and total root weight for various root classes of potato under field grown conditions. Basal roots are important for water uptake and anchorage, whereas stolon roots are connected with nutrient acquisition and tuber formation (Wishart et al., 2013). Despite these efforts, the link between storage root/tuber yield and the carbon partition to other root types as well as the regulatory networks involved in RTCs is yet to be established. Understanding the genetic, physiological and environmental factors influencing these components of RSA in RTCs is therefore critical in adapting genotypes to changing climates.

## Hormonal and Genetic Control Pathways for Root System Architecture

Most genetic studies on RSA have been carried out using ‘model’ plant *Arabidopsis* and a few ‘model’ cereals including maize and rice. In this section we review the hormonal/genetic control of RSA as reported in *Arabidopsis* and/or cereals followed by what is known in RTCs for storage root and tuber development. Formation of LRs as an important aspect of RSA which is a result of cell division of a specific subset of pericyle cells (Casimiro et al., 2001). The cell division process is under the control of both root- and shoot-derived auxin, where cellular levels of auxin contribute to the regulation of gene expression, which then impacts root branching (Overvoorde et al., 2010). Auxin activates *Cyclin-dependent-kinases* (CDKs) (Himanen et al., 2002) and *D-type cyclin* (CYCD) (Nieuwland et al., 2009). These two are cell-cycle genes involved in pericycle division during the LR initiation process and whose inhibition leads to a reduced number of LRs. Also related to the cell cycle initiation is the E2Fa/Dpa transcription factor, which promotes the G1-S transition by controlling the expression of genes required for DNA replication (Boudolf et al., 2004). However, initiation of the cell cycle alone is not enough to initiate LR formation but, as Vanneste et al. (2005) showed, LR initiation requires fine tuning by both negative and positive mechanisms regulating auxin homeostasis and signal transduction in the pericycle. These processes are under the control of auxin-responsive genes dependent on *Auxin/indole-3-acetic acid-auxin response factors* (AUX/IAA-ARFs) auxin signaling pathways. Genes containing auxin-responsive elements (AREs) in the promoter region are directly regulated by ARFs. In the absence of auxin, the ARFs combine with AUX/IAA proteins (AUX/IAA-ARFs) and are therefore not active. In the presence of auxin, however, the AUX/IAA proteins are degraded by auxin-receptor proteins TIR1 and AFBs through the SCF ^TIR1/AFBs^ complexes and 26S proteasomes (Goh et al., 2012). This degradation leaves the ARFs active to either positively or negatively regulate auxin responsive transcription. There are several of these AUX/IAA-ARF modules which are proposed to successively coordinate different developmental processes by regulating distinct targets (De Smet et al., 2010). The exact number of such modules involved in LR development is however still unknown. De Smet et al. (2010) showed a bimodal auxin response where they found that in addition to the *Solitary root/indole-3-acetic acid14 – auxin response factors7 and 19* (SLR/IAA14-ARF7-ARF19), the *Bodenlos/indole-3-acetic acid12/monopteros-auxin response factor5* (BDL/IAA12/MP-ARF5), acting downstream of SLR/IAA14, was required to guarantee organized LR patterning. Goh et al. (2012) listed several modules responsible for different stages of LR initiation, including the IAA28-ARFs module, which regulates the specification of LR founder cells; the SLR/IAA14-ARF7-ARF19, which regulates nuclear migration and asymmetric cell division of the LR founder cells for LR initiation and the BDL/IAA12/MP-ARF5, which regulates LR initiation and organogenesis; the *Short hypocotyl2*/IAA3-ARF (SHY2/IAA3-ARF), which regulates primordia development and emergence after SLR/IAA14-ARF dependent LR initiation, and which also inhibits LR initiation. Each of these modules have target genes. Okushima et al. (2007), for example, showed that the SLR/IAA14-ARF7-ARF19 module regulates LR formation by directly activating *lateral organ boundaries domain/ asymmetric leaves2-like* (LBD/ASL) genes. Many other hormones interact with the auxin signaling pathways during LR initiation Cytokinin (CK) and exogenous abscisic acid (ABA) negatively affect LR development whereas Brassinosteroid (BR) positively affects LR formation. The pericycle founder cell cycling is blocked in the G2 to M transition phase by CK thereby inhibiting LR formation. In the presence of exogenous ABA, emergence of LR primordia from the parent root is inhibited before the LR meristem is activated. Despite this negative regulation of LR development by exogenous ABA, ABA signaling also has cross talks with auxin action via the *ABA insensitive 3* (ABI3) and the *enhanced response to ABA 1* (ERA1) genes which enhance auxin-regulated LR formation. Cross-talk is also indicated between BR and auxin-dependent LR formation, where it is thought to promote acropetal auxin transport (reviewed by Fukaki and Tasaka, 2009). Although most of these studies were carried out in *Arabidopsis*, Orman-Ligeza et al. (2013) compared some of these molecular control pathways in cereals and *Arabidopsis* and found that the AUX/IAA-ARF and the LBD/ASL regulatory pathways were conserved. Several reviews on genetic and hormonal control of RSA are available. Smith and De Smet (2012) have reviewed the genetic control of root branching, giving insights from *Arabidopsis* and cereals. Jung and McCouch (2013) also provide a comprehensive review on the genetic and hormonal control of RSA.

In sweetpotato, the only study found specifically referring to genetic control of RSA is by Villordon et al. (2014a) who showed evidence that orthologs of genes associated with RSA in model crops were present in sweetpotato. They found increased expression of a putative nitrogen transporter and deceased expression of a high affinity nitrogen transporter as well as decreased expression of a MAD-box gene under low nitrogen (N) conditions. A substantial amount of information is however available for storage root formation which is part of RSA in sweetpotato and cassava. Cytokinin is important in regulating storage root development in sweetpotato (Hashizume et al., 1982). *Zeatin Riboside* (ZR), *Trans-Zeatin Riboside* (t-ZR) and *9-glucosyl-n-6-2-isopentenyl adenosine* (i6Ado) are the major CK involved in developing and activating the primary cambium. Besides hormones, several genes are involved in storage root formation and development in sweetpotato. Tanaka et al. (2005) found SRF1 through SRF10 developmentally regulated genes to be involved in storage root formation. SRF1, SRF2, SRF3, SRF5, SRF6, SRF7, and SRF9 were upregulated while SRF4, SRF8, and SRF10 were downregulated during storage root formation. Tanaka et al. (2008) showed that *knotted1-like homeobox* (KNOX1) genes, Ibkn1, Ibkn2 and Ibkn3, are associated with storage root development in sweetpotato. Ibkn1 and Ibkn2 were upregulated in developing and mature storage roots relative to fibrous roots. Ibkn1 is homologous to *shoot meristemless* (STM) gene of *Arabidopsis* whose overexpression leads to higher CK levels, while Ibkn2 and Ibkn3 are homologous to *Brevipedicellus* gene of *Arabidopsis* which negatively regulates lignin biosynthesis. A group of MAD-box genes, IbMADS genes such as IbMADS3, IbMADS4, and IbMADS79 are also found in fibrous roots before thickening, mainly in the vascular cambium region where rapid cell division occurs during storage root thickening (Kim et al., 2002). Noh et al. (2010) found that a MADS-box protein copy DNA, SRD1 enhances the proliferation of the metaxylem and cambium cells during the auxin-dependent initial thickening and growth of storage roots. Storage root development in sweetpotato is enhanced when an expansin gene (IbEXP1) is down-regulated (Noh et al., 2012), but lignin biosynthesis is inhibited as starch biosynthesis is enhanced during early storage root formation (Firon et al., 2013). Details on the molecular regulation of storage root formation in sweetpotato have been reviewed by Ravi et al. (2009, 2014). No literature was available on the genetic control of ARs and LRs in cassava. However, de Souza et al. (2004) showed overexpression of the Mec1 gene which codes for a Pt2L4 glutamic acid-rich protein and a RING Zinc Finger and LEA protein genes in the secondary xylem tissue of storage roots relative to fibrous roots. Based on a correlation network, the relationship between KNOX1 genes, phytohormone biosynthesis and phytohormone-signaling genes was established, and it was hypothesized that phytohormones are involved in the initiation of storage root development in cassava (Sojikul et al., 2015). Both potato and cassava storage organs have been substantially studied, but not the genetic and hormonal control of RSA for either crop.

In addition to hormones, signaling components, and transcription factors, micro-interfering RNAs (miRNAs) and small-interfering RNAs (siRNAs) have been shown to affect RSA in plants, as reviewed by Meng et al. (2010) and Khan et al. (2011). The miRNAs and siRNAs are thought to be involved in auxin signaling, nutrition metabolism and stress response by mediating signal interactions. They have been identified in embryonic root development, radial patterning, formation of ARs and LRs. However, their role in RTCs has not yet been studied.

## The Relationship Between Root System Architecture and Abiotic Stresses

Root system architecture has a central role in crop plants’ response to abiotic stresses. Since roots grow underground, they are the first to sense abiotic stresses and adjust their genetic program for post-embryonic development to survive the stress (Lynch, 1995). Plant roots obtain water and nutrients from the soil, which is a complex system with intrinsic properties, abiotic and biotic interactions. Modulation of RSA is therefore affected when changes in the plant nutritional status and external nutrient supply over time are perceived and integrated into the intrinsic root development program. The degree of root plasticity is based on variations in the number, extension, placement, and growth direction of individual components of the root system (Giehl et al., 2014). These changes in RSA consequently affect the growth and development of above-ground biomass (Paez-Garcia et al., 2015) by altering carbon allocation to shoots and/or triggering signaling pathways involving hormones, proteins, RNAs, among others (DoVale and Fritsche-Neto, 2015). In this case therefore, roots indirectly regulate leaf stomatal conductance and affect leaf blade posture and photosynthetic rate when exposed to abiotic stress.

Different abiotic stresses affect RSA in varied ways. **Table 2** summarizes the root traits necessary for adaptation to different abiotic stresses. Deeper roots are associated with increased acquisition of water and mobile nutrients like N that may leach to lower soil layers (Lynch and Wojciechowski, 2015). LRs, the main determinants of ultimate RSA, are influenced strongly by moisture and nutrient distribution in the soil (Postma et al., 2014). Deak and Malamy (2005) showed that LR formation from LR primordia in *Arabidopsis* is repressed under drought stress when ABA and *lateral root development* (LRD2) gene, interact with auxin. Since ABA, LRD2 and auxin are also involved in RSA even without drought stress, it appears that such genes like LRD2 regulate the formation of LRs through promotive and repressive hormone signaling pathways depending on the environmental conditions. Repression of LR development under abiotic stress is of particular importance in root crops. In sweetpotato for example, the final storage root yield depends on the capacity of a genotype to develop LRs on the main ARs. Those with arrested or non-emerged LRs develop lignified steles, which inhibit localized swelling into storage roots (Villordon et al., 2012). Other Important contributors to RSA include single-cell projections from root epidermal cells referred to as root hairs (Tanaka et al., 2014). A high density of both root hairs and LRs is associated with increased nutrient uptake, especially in the top soil (Postma et al., 2014) but increased metabolic costs is a trade-off here (Zhan et al., 2015). There are other trade-offs associated with crop adaptation to individual abiotic stresses. Primary root length is inhibited under low-soil phosphorus (P) conditions, while LR development is promoted thereby leading to a shallower root system. This has negative effects under drought stress where deeper roots are necessary in order to have better access to water (Wasson et al., 2012). Reduced frequency of LR branching improves N uptake where genotypes with fewer but longer LRs have greater axial root elongation, deeper roots and better N uptake than those with a higher number of LRs (Zhan and Lynch, 2015). On the other hand, a larger number of LRs is required under P-limited conditions for topsoil foraging (Lynch and Brown, 2001). Since abiotic stresses normally occur in combination under field conditions, it is therefore evident that there is ‘no size fits all’ if adaptation to abiotic stress conditions is done considering each stress individually.

**Table 2:** A summary of relevant phenotypes and required traits under different abiotic stresses.

	Desired phenotypes	Required traits	Ref. general	Ref. RTCs
Drought	• Deeper root systems • Redistribution of branch root density from surface to depth • Increased radial hydraulic conductivity at depth • Reduced metabolic costs	• Longer primary roots • Larger root tip diameter • Steeper, abundant and longer lateral roots • Reduced cortical cell file number • Larger root cortical aerenchyma • Gravitropism	•Wasson et al., 2012 •Uga et al., 2013 •Lynch, 2013 •Lynch et al., 2014 •Comas et al., 2013	•Wishart et al., 2014 • Pardales and Yamauchi, 2003
Nutrient deficiency	• Top soil foraging • Rhizosphere modification • Reduced metabolic costs	• Abundant and longer root hairs • Abundant and longer lateral roots • Shallow and abundant adventitious roots • Exudation of organic anions • Association with microbes • Larger root cortical aerenchyma • Reduced root respiration	• Lynch and Brown, 2001 • Richardson et al., 2009 •Forde, 2014 •Gruber et al., 2013 •Lynch, 2015 • Walch-Liu et al., 2006 • Postma and Lynch, 2011 •Nielsen et al., 1998 •Nielsen et al., 2001	• Melteras et al., 2008 •Hgaza et al., 2012 •O’Sullivan, 2008 •Wishart et al., 2013
Salinity	• Water extraction efficiency • Ion exclusion	• Reduction in main root elongation • Redistribution of root mass between main and lateral roots • Reduction in sodium transport to shoots • Compartmentalization of sodium ions into the root steles and vacuoles	• Munns and Tester, 2008 • Julkowska et al., 2014 •Roy et al., 2013 •Rus et al., 2006 •Katori et al., 2010 • Gupta and Huang, 2014	• None

In RTCs, it is known that the root system is made up of ARs and LRs which presumably are involved in water and nutrient uptake and hence respond to abiotic stress. However, some RTCs have complex RSA because the harvestable part is also underground with several root classes, e.g., in potato, which may have different functions with regard to adaptation to abiotic stress. The potato root system is known to be shallow, with poor ability to penetrate soils thereby being drought susceptible (Porter et al., 1999). Despite having a shallow root system, potato is still not efficient in P and N uptake because the larger root system has a respiration carbon cost (Balemi and Schenk, 2009). Furthermore, most findings studied the root system as a whole without identifying possible roles for different root classes. An attempt at this was done by Wishart et al. (2013) who reported genetic variation for potato root traits without any specific abiotic stress. They suggested that basal roots were responsible for water uptake and anchorage while stolon roots were responsible for nutrient uptake and tuberization. Cassava and sweetpotato have less root classes compared to potato because the harvestable part is also a root. Pardales et al. (1999) studied the effects of high root zone temperature on root systems of cassava and sweetpotato. They showed a reduction in the total length of ARs, number and total length of first order LRs, under high root zone temperature in both crops. Pardales and Yamauchi (2003) also showed a suppression of AR and LR formation and development under drought stress in both sweetpotato and cassava. Recent studies in sweetpotato indicate direct influence on RSA of spatial and temporal availability of water and N availability similar to model systems (Villordon et al., 2014a). In yam, Hgaza et al. (2012) found no fertilizer response of tuber yield but a positive correlation between thinner, longer roots with tuber yield.

## Manipulating Root System Architecture for Abiotic Stress Tolerance

The aim of carrying out RSA studies in crop plants is to understand areas of interest within the root system and incorporate this information in crop improvement programs. Several approaches may be applied in manipulating RSA in order to adapt crops to changing climates.

### Combined-Stress versus Single-Stress Selection

Most of the reported studies above were carried out for stress-specific responses. However, stresses always occur in combination, with different scenarios and complexity, to which responses are also varied. In this case therefore, combined stress scenarios may be considered in relation to RSA. The complexity of the root growing environment and the limitations associated with studying only one trait could be lessened by ideotype or trait-based breeding, originally proposed by Donald (1968). Ideotype breeding requires consideration of relationships between multiple traits in addition to each individual trait (Rasmusson, 1987). A potato root ‘ideotype’ as summarized by Wishart et al. (2013) for example, is hypothesized to be one which is architecturally plastic, long, deep, and thin, as well as being able to self-protect against parasites and pathogen. Such a system should ideally be responsive to water deficits, able to transport ABA to shoots, enable efficient stomatal closure, and have minimum carbon costs, either by producing aerenchyma or increasing specific root length, increasing root surface area, or desirable root exudates and symbioses in the rhizosphere. In sweetpotato, Villordon and Clark (2014) found an association among RSA, virus resistance and availability of N thereby indicating the necessity of a more systematic approach toward determining and managing yield constraints. They showed that AR and LR formation was decreased by about 51% under deficiency and virus infection. On the other hand, storage root formation is known to fail under excess moisture and high N as more biomass is partitioned to above-ground biomass (Villordon et al., 2012). An ideotype for sweetpotato therefore, in addition to being efficient in water and mineral uptake and utilization, and being pathogen resistance as indicated above for potato, should also be tolerant to excess moisture and have balanced biomass partitioning between storage root and above-ground biomass. Trait -based breeding does not always result in accumulation of additive effects, rather synergies among traits need to be considered. It is proposed to incorporate root phenes into breeding programs targeting RSA. A phene, which has variants referred to as phene states, is the basic functional unit of a phenotype, with a phenotype being defined as the particular combination of states of all phenes of an individual (York et al., 2013). With regard to RSA, Lynch and Brown (2012) propose ‘elementary’ and ‘unique’ root phenes which cannot be decomposed further and which are a result of only one set of genes and processes. Root growth angle may for example be considered one of the phenes of root depth as it is only one of the factors determining root depth, while root depth is referred to as a phene aggregate, being a result of several phenes. A group of interacting phenes that may be selected together are referred to as a phene module. York et al. (2013) proposed hypotheses for integrating root phenes in a breeding program. They considered synergies within a phene module which increase as the number of positively acting phene-state combination increases. Metabolic costs are to be expected with such synergies except in metabolically neutral, positively acting, phene-state combinations. The interactions between phenes within plants, between plants and with the environment are expected to result in genetic variation in RSA.

### Model-Assisted Phenotyping

In breeding approaches such as ideotype or phene-integrated, structural-functional plant modeling and simulation may offer a robust way of understanding the complexity of the non-linear signals and transduction pathways involved in the roots’ responses to multiple abiotic stresses. This is expected to provide new mechanistic insights into the regulation of root growth and development (Chickarmane et al., 2010). In addition to advances in high-throughput phenotyping techniques that allow rapid evaluation of a large number of genotypes, model-assisted phenotyping enhances prediction of difficult traits such as those that vary with environmental conditions. It also allows precise prediction of genotype × environment × management interaction over a large number of environments thereby allowing the estimation of comparative advantage of a given phene state in different environments (Tardieu and Tuberosa, 2010). Multi-scale modeling which examines behavior at subcellular, cellular, tissue, organ, and whole organism states may allow the prediction of the effect of a given phene, phene state, phene module, or phenotype in a complex abiotic stress environment (Band et al., 2012). Leitner et al. (2014) showed that functional-structural root models were appropriate to better comprehend the role of roots in whole-plant adaptation to diverse drought scenarios, in addition to their contribution to distinct drought scenarios. Using a dynamic root architecture model and root xylem hydraulic properties model, they showed that plants which transpired more had root axes which matched the available water distribution. They also found that water saving genotypes had lower root conductance than the water spending genotypes. Despite these advantages, encouragement for the adoption of root models needs to be accompanied with realistic and more explicit plant regulatory networks, in addition to integration with phenomic databases (Dunbabin et al., 2013) in order to be more representative and applicable to actual field performance of genotypes. This approach has not been applied in RTCs yet.

### Genomics-Based Approaches

Manipulating root traits has been carried out in several crops through the use of molecular markers. Several specific genes related with RSA were identified in crop plants, either from gene mutants with quantifiable characteristics, or from QTL analyses. The genetic control of LR formation as reviewed above was elucidated based on gene mutants with quantifiable characteristics. In rice, a gene controlling root angle, *Deeper Rooting 1* (DRO1), was identified using QTL mapping and introgressed into an elite line through backcrossing, whereas *Phosphorus Starvation 1* (PSTOL1), a *pup-1*-specific protein kinase gene confers early root growth for P-acquisition in rice (Gamuyao et al., 2012) and sorghum (Hufnagel et al., 2014). However, information on how these genes/QTL affect the phenotypes and/or their performance in different genetic backgrounds and/or different environments is still largely lacking. This is because RSA response to environmental conditions is normally quite different under field conditions, given the broader spectrum of stresses the roots find themselves in Rich and Watt (2013). Extrapolating results obtained from a response to a specific abiotic stress is not therefore adequate (Jung and McCouch, 2013). Most of these QTLs are small-effect QTLs, i.e., they are normally conditioned by a single gene and therefore not stable across environments. Kitomi et al. (2015) for example, established that genotypes having the same functional allele of DRO1 could have different rooting angles. Small-effect QTLs therefore are assumed to be part of a set of minor QTLs. In such cases, it is necessary to carry out comparative data analysis and integration across controlled environments and field studies to establish target variants for further investigation and introgression into genotypes of interest. Alternatively, identification of large-effect QTLs which are more stable across environments and genetic backgrounds seems to be the most promising way of ensuring impact from genomics-assisted breeding methods. Dixit et al. (2015) confirmed the multi-genic and multi-environment effectiveness of qDTY_12.1_, a large-effect QTL identified on chromosome 12 of the rice genome. They confirmed the central role of the *no apical meristem* (OsNAM_12.1_) transcription factor in the activity of qDTY_12.1_ together with promoters of six intra-QTL genes with NAM binding sites as well as three co-localized and/or partially co-expressed genes of OsNAM*_12.1_*. These findings suggested that identification and proper analysis of large-effect QTLs together with their component genes could lead to a more efficient breeding process for complex traits such as those involved in adaptation and abiotic stress tolerance.

These reports are mainly based on *Arabidopsis* and cereal crops with simple genetic make-ups. Most RTCs on the other hand are polyploid with very complex genetic backgrounds. Genetic analysis of these crops is complicated by multiple alleles and loci, mixed inheritance patterns, association between ploidy and variation in mating system, among others (Dufresne et al., 2014). Marker-based procedures developed in diploid species therefore present difficulty to apply in most polyploid RTCs and adoption of these approach is not therefore straight forward. Application of the most commonly used genotyping methods, including new generation sequencing techniques, in RTCs present problems in allele dosage determination, presence of null alleles, distinguishing orthologs from paralogs, and copy number variation (Dufresne et al., 2014). As a consequence, although new techniques such as genomic selection offer great potential in marker-based breeding, they are currently still difficult to adopt. Additive, dominant and epistatic genetic effects are all important in RTCs due to heterozygosity (Ceballos et al., 2015) while models developed for genomic selection in cereals mainly consider additive genetic effects. Marker-based approaches in this class of crops therefore require re-thinking on the methods and pipelines available so far.

In addition to identifying QTLs and genes of interest from a species, another approach for adapting roots to abiotic stress is through transgenic technology. For example, *spermidine synthase* genes (FSPD1) confer higher antioxidant enzyme activities to plants. Under abiotic stress, plants with higher antioxidant enzyme activities are generally more tolerant, as they are better able to remove by-product reactive oxygen species (ROS) that are harmful to the plant if allowed to accumulate. Using transgenic technology, sweetpotato transformation with *spermidine synthase* genes (FSPD1) from *Cucurbita ficifolia* increased their multiple stress tolerance, with a higher concentration of FSPD1 in leaves and storage roots (Kasukabe et al., 2006). Estrada-Melo et al. (2015) used a *9-cis-epoxycarotenoid dioxygenase* gene from tomato (LeNCED1) overexpressed in petunia and confirmed that NCED increased drought resistance of the transgenic plants. A calcium-dependent protein kinase (OsCDPK7) conferred tolerance to cold and salt/drought in rice transgenics. Two distinct pathways for cold and salt/drought tolerance using a single CDPK were implied which showed that manipulation of CDPK has great potential to adaptation and abiotic stress tolerant crop improvement. ‘Gene stacking,’ a form of ideotype breeding, could be a good alternative to transgenic technology that relies on a single gene. However, this has only been successful in pest control engineering such as the *Bt* toxin resistance (York et al., 2013). Gene stacking for complex traits is therefore still a challenge due to trait interaction.

Genome editing, a new approach that involves targeted DNA sequence modification through creation of double-strand breaks using sequence specific nucleases, provides possibilities to change a protein’s amino acid sequence through specific nucleotide substitutions, delete genes or chromosome segments, and introduce foreign DNA at desired genomic regions (Voytas, 2013). Several nucleases are available for targeted genome engineering (reviewed by Esvelt and Wang, 2013), but the system receiving most attention recently is CRISPR/Cas9, which involves the use of a guided RNA to create targeted mutations in candidate genes of key pathways in order to identify their effects and create new variation within a relatively short time (Cong et al., 2013), among other potential uses. These methods are gaining application in crop plants including RTCs. Clasen et al. (2016) used a TALENs approach to improve cold storage and processing traits in potato. The *vacuolar invertase* (VINV)gene (VINV) encoding a protein that breaks down sucrose to glucose and fructose was silenced in order to minimize the accumulation of reducing sugars which turn into anti-nutrients upon processing.

No matter which manipulation method is followed, proper phenotypic evaluation prior to and after such manipulations is important in order to realize and quantify genetic gains from RSA manipulation in a breeding program.

## Phenotyping Root System Architecture Traits: Available Technologies/Challenges

Since roots grow below ground, studying the entire root system naturally requires digging it out, a complex process in itself, and it is difficult to extract the entire system without breaking off the finer parts. For this reason, studies have mainly dwelled on above-ground traits related to abiotic stress tolerance. However, given the pressures on crop productivity caused by global climate change, with the associated abiotic stresses, and the notion that food production needs to double in the next few years to accommodate the growing global population, root manipulation seems to hold the key toward sustainable food production. Villordon et al. (2014a) suggested that a paradigm shift toward RSA studies would enable a truly inclusive green revolution and allow food-insecure, resource-poor farmers who depend on RTCs in developing countries to also benefit. With this mindset, plant biologists, geneticists, and breeders have now shifted some focus toward studying of root traits. Due to the aforementioned complexity of studying roots under the soil, plant scientists are now set on finding minimally intrusive, non-destructive, whole-root system evaluating platforms. Hydroponics and gels are the most widely used systems to phenotype root systems (Jung and McCouch, 2013). Although, they offer a simple way to study different root traits and have given insight into root growth and development, both are controlled and do not represent actual field environments, and correlation of the findings from such experiments with actual performance of a plant in its natural environment are limited. To address this bottleneck, plant scientists are continually seeking to develop methods that will allow study of RSA in a more natural environment. Several methods have been proposed and applied in various studies including ‘shovelomics’ (Trachsel et al., 2011), soil coring (Wasson et al., 2014), rhizolysimeters (Eberbach et al., 2013) and minirhizotrons (Maeght et al., 2013), which are all soil-based. However, these methods are also low throughput, slow, and not amenable to large numbers of genotypes like those required for genome-wide association mapping studies. Image-based systems have also been developed and proposed to study roots in their natural environments, including X-ray computed tomography (Tracy et al., 2010) where x-rays are used to acquire 3-D cross-sectional images of the roots within the soil, Laser (Braga et al., 2009) which allows collection of bio-speckle patterns of gel-grown roots, nuclear magnetic resonance (NMR: Menzel et al., 2007), ground penetrating radar (GPR; Hirano et al., 2012), infra-red (IR) imaging (Dokken and Davis, 2007), and near-infra-red (NIR) imaging (Tirlapur and König, 1999), among others. However, application of some of these methods is still limited by the costs involved and to a few genotypes. Another bottleneck associated with imaging methods is image analysis. Several root image analysis platforms have been developed to address this limitation^2^. With these large numbers of imaging and image analysis platforms, the need for sharing and use of data requires establishment of trait ontology across them to allow development of root ideotypes for different environments. Efforts by Lobet et al. (2015) to develop a unified root architecture development language are therefore right on time. This, combined with scaling up of the image analysis methods mentioned above, will be able to provide further knowledge required to adapt crops to their highly variable environments.

## Conclusion

The increasing global population requires increased food production on the same or even less agricultural land as used currently, if the effects of climate change render some of the available marginal lands unfit for agricultural production. Most of the present and past crop improvement efforts have focused on above-ground traits to adapt crop plants to different production constraints. Although great progress has been made, and food production significantly increased, by manipulating above-ground traits, an estimated 800 million people are still food insecure, whereas yields, especially in cereal, have reached their yield potential and are plateauing in certain regions of the globe. It is therefore time for crop scientists to tap into un-explored and less exploited diversity within RSA traits to ensure rapid genetic gains, and stable and enhanced productivity of agricultural systems for future environmental conditions and climate change scenarios. Due to the quantitative nature of RSA traits and complex interaction of several underlying pathways that control them, response of RSA to multiple individual stresses or combination of stresses is variable. Modeling of the responses of root traits to multiple stress scenarios in a combination of high-throughput root-trait phenotyping techniques, alongside a robust database and data analytical pipeline, could be a way to go. This proposed strategy is applicable to all crops, but is more urgent in RTCs, as the second largest source of food security after cereals, mainly growing in marginal areas where many cereals cannot survive. Also, for RTCs, the harvestable organs are part of the RSA. It is recommended to increase focus on RSA research by investing more resources. RTCs can learn from what has been found so far in cereals and adopt some of their methods, while developing high-throughput techniques to quantify RSA traits under optimal and stressful conditions.

## Author Contributions

Review was conceptualized and written by MK, DG, and AV.

## Conflict of Interest Statement

The authors declare that the research was conducted in the absence of any commercial or financial relationships that could be construed as a potential conflict of interest.

## Footnotes

1 http://www.fao.org/docrep/x5415e/x5415e01.htm

1 www.plant-image-analysis.org
